# The PneuCarriage Project: A Multi-Centre Comparative Study to Identify the Best Serotyping Methods for Examining Pneumococcal Carriage in Vaccine Evaluation Studies

**DOI:** 10.1371/journal.pmed.1001903

**Published:** 2015-11-17

**Authors:** Catherine Satzke, Eileen M. Dunne, Barbara D. Porter, Keith P. Klugman, E. Kim Mulholland, PneuCarriage project group

**Affiliations:** 1 Pneumococcal Research Group, Murdoch Childrens Research Institute, Royal Children’s Hospital, Parkville, Victoria, Australia; 2 Department of Microbiology and Immunology, University of Melbourne, Peter Doherty Institute for Infection and Immunity, Parkville, Victoria, Australia; 3 Hubert Department of Global Health, Rollins School of Public Health, Emory University, Atlanta, Georgia, United States of America; 4 Department of Infectious Disease Epidemiology, London School of Hygiene & Tropical Medicine, London, United Kingdom; Imperial College London, UNITED KINGDOM

## Abstract

**Background:**

The pneumococcus is a diverse pathogen whose primary niche is the nasopharynx. Over 90 different serotypes exist, and nasopharyngeal carriage of multiple serotypes is common. Understanding pneumococcal carriage is essential for evaluating the impact of pneumococcal vaccines. Traditional serotyping methods are cumbersome and insufficient for detecting multiple serotype carriage, and there are few data comparing the new methods that have been developed over the past decade. We established the PneuCarriage project, a large, international multi-centre study dedicated to the identification of the best pneumococcal serotyping methods for carriage studies.

**Methods and Findings:**

Reference sample sets were distributed to 15 research groups for blinded testing. Twenty pneumococcal serotyping methods were used to test 81 laboratory-prepared (spiked) samples. The five top-performing methods were used to test 260 nasopharyngeal (field) samples collected from children in six high-burden countries. Sensitivity and positive predictive value (PPV) were determined for the test methods and the reference method (traditional serotyping of >100 colonies from each sample).

For the alternate serotyping methods, the overall sensitivity ranged from 1% to 99% (reference method 98%), and PPV from 8% to 100% (reference method 100%), when testing the spiked samples. Fifteen methods had ≥70% sensitivity to detect the dominant (major) serotype, whilst only eight methods had ≥70% sensitivity to detect minor serotypes. For the field samples, the overall sensitivity ranged from 74.2% to 95.8% (reference method 93.8%), and PPV from 82.2% to 96.4% (reference method 99.6%). The microarray had the highest sensitivity (95.8%) and high PPV (93.7%). The major limitation of this study is that not all of the available alternative serotyping methods were included.

**Conclusions:**

Most methods were able to detect the dominant serotype in a sample, but many performed poorly in detecting the minor serotype populations. Microarray with a culture amplification step was the top-performing method. Results from this comprehensive evaluation will inform future vaccine evaluation and impact studies, particularly in low-income settings, where pneumococcal disease burden remains high.

## Abstract 2

Catherine Satzke and collaborators exploit a panel of spiked and field pneumococcal samples to compare serotyping methods used for nasopharyngeal carriage studies.

## Editors' Summary

**Background:**

About 800,000 young children, mostly living in low-income countries, die annually from pneumococcal diseases, illnesses caused by the *Streptococcus pneumoniae* bacterium. *S*. *pneumoniae* is transmitted through contact with infected respiratory secretions and harmlessly colonizes the nose and throat of many healthy children (nasopharyngeal and oropharyngeal carriage). Occasionally, however, *S*. *pneumoniae* spreads into the lungs, the blood stream, or the covering of the brain, where it causes pneumonia, septicemia, and meningitis, respectively. These potentially fatal invasive pneumococcal diseases can be treated with antibiotics but can also be prevented by vaccination. Vaccination primes the immune system to attack disease-causing organisms (pathogens) by exposing it to weakened or dead pathogens or to pathogen molecules that it recognizes as foreign (antigens). Because there are more than 90 *S*. *pneumoniae* variants, or “serotypes,” each characterized by an immunologically distinct complex sugar coat, *S*. *pneumoniae* vaccines have to include multiple serotypes. “Pneumococcal conjugate vaccines” (PCVs) effectively prevent invasive pneumococcal diseases caused by common serotypes in resource-rich countries and are now being introduced into resource-poor countries.

**Why Was This Study Done?:**

Although vaccination with PCVs reduces the carriage of the pneumococcal serotypes contained in them (vaccine serotypes), in the absence of these serotypes, non-vaccine serotypes can rapidly colonize the nasopharynx and become more common in both carriage and disease. Thus, “serotype replacement,” which may be more pronounced in low-income settings, threatens the global control of pneumococcal disease through vaccination; thus, when evaluating the impact of pneumococcal vaccines, it is important to analyze the carriage of multiple serotypes. Unfortunately, the traditional serotyping method—in which bacterial colonies are grown and a small number of colonies are typed using tests called the Quellung reaction and latex agglutination—is cumbersome and frequently misses the carriage of multiple serotypes. Several new serotyping methods have been developed over the past decade, and, here, in a multi-center comparative study (the PneuCarriage project), the researchers investigate which of these new methods is best for the examination of pneumococcal carriage in vaccine evaluation studies.

**What Did the Researchers Do and Find?:**

The researchers used 15 clinical isolates containing a mixture of pneumococcal serotypes to prepare 81 “spiked” samples, which they distributed to 15 laboratories for testing with 20 serotyping methods. They determined the sensitivity (the percentage of serotypes in the samples that were correctly identified) and the positive predictive value (PPV; the proportion of identified positives that were true positives) for each method and used the five top-performing methods (those with the highest sensitivity and PPV; a perfect test has a sensitivity and a PPV of 100%) to test 260 nasopharyngeal (field) samples collected from children in six high-burden countries. When testing the spiked samples, traditional serotyping of over 100 colonies per sample had a sensitivity of 98% and a PPV of 100% overall, whereas the sensitivity of the alternative methods ranged from 1% to 99%, and their PPV ranged from 8% to 100%. Fifteen methods detected the major serotype in the spiked samples with ≥70% sensitivity, but only eight detected the minor serotypes with the same sensitivity. For the field samples, the sensitivity and PPV of the top-performing tests ranged from 74.2% to 95.8% and from 82.2% to 96.4%, respectively (the sensitivity and PPV of the traditional method were 93.8% and 99.6%, respectively); a culture microarray method had the best overall performance (95.8% sensitivity and 93.7% PPV).

**What Do These Findings Mean?:**

A pneumococcal serotyping method for use in carriage studies needs to have high sensitivity, to detect multiple serotypes in individual samples, and to detect most or all serotypes. These findings show that although most of the recently developed serotyping methods detected the dominant serotype in a sample, many failed to detect minor serotypes. Moreover, the performance of similar methods varied markedly, and methods optimized for testing pure isolates did not necessarily work well when testing more complex samples. These findings identified microarray with a culture amplification step as the top-performing method, but, importantly, this study did not test all the available serotyping methods. Also, because it assessed each method in only a single laboratory, no conclusions can be reached about the reproducibility of these methods or their suitability for use in less-experienced laboratories or in resource-limited settings. Nevertheless, these findings should help to guide future vaccine evaluation and impact studies, particularly in low-income settings, where the burden of pneumococcal disease remains high.

**Additional Information:**

This list of resources contains links that can be accessed when viewing the PDF on a device or via the online version of the article at http://dx.doi.org/10.1371/journal.pmed.1001903.

- The US Centers for Disease Control and Prevention provides information on all aspects of pneumococcal disease and pneumococcal vaccination, including personal stories (including some information in Spanish)
- The UK National Health Service Choices website provides information about pneumococcal disease
- Kidshealth, a website provided by the US-based not-for-profit Nemours Foundation, includes information on pneumococcal vaccination (in English and Spanish)
- Public Health England provides guidance on pneumococcal disease and vaccination
- The not-for-profit Immunization Action Coalition has information on pneumococcal disease, including personal stories
- Gavi, a not-for-profit global vaccine alliance, is helping to roll out pneumococcal vaccination in resource-poor countries
- More information about the PneuCarriage project is available
- MedlinePlus provides links to other resources about pneumococcal infections (in English and Spanish)
- The World Health Organization provides information about pneumococcal disease and pneumococcal vaccines.

## Introduction

*Streptococcus pneumoniae* (the pneumococcus) is a dominant cause of childhood illness and death worldwide. It has been estimated to cause ~800,000 deaths in children aged under 5 y annually, most due to pneumonia and most in low-income countries [1]. Pneumococci commonly colonise the nasopharynx of healthy children. Although carriage is usually asymptomatic, it can cause local inflammation and is considered a prerequisite for pneumococcal disease [2]. Pneumococci are diverse, with 97 serotypes identified to date. Understanding carriage is important for understanding pneumococcal population biology and transmission, assessing vaccine impact, and evaluating the performance of new vaccines.

Pneumococcal conjugate vaccines (PCVs) are effective at preventing pneumonia and invasive pneumococcal disease, and are currently being introduced in many resource-poor countries (e.g., by Gavi, the Vaccine Alliance). PCVs reduce nasopharyngeal carriage of the pneumococcal serotypes they contain (vaccine types). Although transmission and disease due to vaccine types is reduced, non-vaccine types fill the ecological niche, becoming more common in carriage and disease [3–5]. This phenomenon of serotype replacement, which may be more pronounced in low-income settings (because of higher rates, load, and diversity of pneumococcal carriage [6] and higher rates of disease), represents a significant risk to the global pneumococcal immunisation strategy. It is difficult to monitor the serotypes causing pneumonia or invasive disease because of the insensitivity of blood culture for detecting pneumococcal pneumonia, inconsistencies in clinical practice (e.g., performing of lumbar punctures), and limitations on diagnostic laboratory capacity, particularly in resource-poor settings [7–9]. Furthermore, any improvement in laboratory or clinical diagnostic practices over time in vaccinated populations would lead to enhanced detection of non-vaccine types. This increase may not reflect true replacement, and may cause unnecessary concerns about vaccine effectiveness and inappropriate changes to vaccine policy.

Carriage studies offer a practical approach for monitoring serotype replacement and can help in assessing the impact of PCVs and other pneumococcal vaccines [8,10]. Pneumococcal carriage is an important endpoint for efficacy trials of new pneumococcal vaccines such as common protein, combination (PCV + common protein), and whole-cell vaccines [11]. Traditional serotyping methods involve typing a small number of colonies, frequently missing carriage of multiple serotypes and providing no quantitative data. The gold-standard serotyping method (the Quellung reaction) was developed in the early 1900s (see [12]) and is performed by testing colonies with a set of antisera and visualising the bacteria with a microscope. It is laborious and requires a complete set of type-specific antisera, and is therefore mainly performed by reference laboratories. Alternate serotyping methods have been developed, but few data formally comparing the performance of these methods to the gold-standard Quellung reaction, or to each other, are available [13].

A pneumococcal serotyping method suitable for use in carriage studies should have high sensitivity (including the ability to detect multiple serotypes), detect most or all serotypes, be suitable to scale up for large projects, and be practical for resource-poor countries. We established the PneuCarriage project, a large, international multi-centre study, with the aim of identifying the best pneumococcal serotyping methods for carriage studies.

We compared 20 different pneumococcal serotyping methods from 15 research groups using 81 laboratory-prepared samples. We then tested the five top-performing methods using 260 nasopharyngeal samples collected from children in six high-burden low- and middle-income countries. All samples were subjected to comprehensive conventional microbiology to thoroughly characterise the pneumococcal content, and the results were used to assess the performance of the new methods to identify the best pneumococcal serotyping methods. Sensitivity and positive predictive value (PPV) were selected as the key parameters for method evaluation, as these measures best convey the overall ability of a method to correctly detect pneumococcal serotypes present in a sample.

## Methods

All research involving human participants was approved by the relevant institutional review board or an equivalent committee, specifically the Bangladesh Institute of Child Health Ethics Review Committee; the Fiji National Research Ethics Review Committee; the Gambian Government/Medical Research Council Laboratory Joint Ethics Committee and the Medical Research Council (UK) Gambia Scientific Coordinating Committee; the Kenya Medical Research Institute National Ethical Review Committee and Scientific Steering Committee; the Government of Papua New Guinea Medical Research Advisory Committee and the Papua New Guinea Institute of Medical Research Institutional Review Board; the Human Research Ethics Committee of the University of the Witwatersrand, the Ethics Committee of Stellenbosch University, and the Medicine Control Council of South Africa; and the Clinical Science Review Committee of the Division of AIDS, US National Institutes for Health. Written informed consent was obtained from the parents/guardians of all participants.

### Spiked Samples

Fifteen pneumococcal isolates were used to prepare 81 laboratory-prepared (“spiked”) samples in skim milk-tryptone-glucose-glycerol (STGG) medium [14,15]. The 15 isolates used to construct the spiked samples were derived from four geographical areas (Bangladesh, Fiji, South Africa, and the United States). Isolates were selected following a review of the literature, and the selection was informed by our international steering committee. The selection included serotypes both common and rare in carriage and a mix of vaccine and non-vaccine types (Table 1). Fourteen of the isolates were derived from the nasopharynx. The serotype 5 isolate was sourced from invasive disease (site not known) because of its relative rarity in carriage. Isolates were serotyped by latex agglutination and Quellung reaction. Quellung reactions were independently confirmed by the Microbiological Diagnostic Unit, University of Melbourne, Australia. Multilocus sequence typing (MLST) was performed as previously described [16]. All isolates had a >80% survival (mean 96.8% [95% CI: 91.1%, 102.5%]) and were able to be serotyped by Quellung reaction and latex agglutination when cultured following a freeze–thaw step. To create the spiked samples, fresh overnight pneumococcal cultures were recovered from horse blood agar (HBA) plates, resuspended in physiological saline, and used to inoculate 5 ml of STGG medium. Once inoculated, tubes were immediately placed at ultra-low temperature (ULT) (≤−70°C) for ≥24 h. Spiked samples were then thawed, vortexed, and dispensed into 60μl aliquots in 0.5ml screw-capped tubes. Aliquots were immediately frozen at ULT and held at that temperature until use (≥24 h).

**Table 1:** Isolates used to create spiked samples.

Isolate	Site of Isolation	Country of Origin^1^	Serotype by Quellung Reaction	Multilocus Sequence Type (ST)	Year of Sample Collection	Child Age at Sample Collection (in Months)
PMP812	Invasive^2^	Bangladesh	5	289	2006	3
PMP825	Nasopharynx	Bangladesh	12F	10232	2005	3
PMP817	Nasopharynx	Bangladesh	15A	6332	2006	3
PMP818	Nasopharynx	Bangladesh	20	5392	2006	3
PMP492	Nasopharynx	Fiji	6B	4781	2007	17
PMP847	Nasopharynx	Fiji	8	404	2007	17
PMP284	Nasopharynx	Fiji	29	9987	2006	18
PMP241	Nasopharynx	South Africa	4	5410	2005	9
PMP228	Nasopharynx	South Africa	6A	1447	2007	7
PMP221	Nasopharynx	South Africa	23F	242	2007	7
PMP219	Nasopharynx	South Africa	14	10231	2007	7
PMP849	Nasopharynx	United States	1	227	2007	363
PMP846	Nasopharynx	United States	9V	1269	2007	7
PMP843	Nasopharynx	United States	19F	3040	2006	66
PMP845	Nasopharynx	United States	38	10230	2006	94

^1^Isolates were kindly provided by Prof. Samir Saha (Bangladesh), Assoc. Prof Fiona Russell (Fiji), Dr. Peter Adrian and Prof. Shabir Madhi (South Africa), and Prof. Kate O’Brien (United States).

^2^Site of isolation not known.

Viable counts were performed in saline to assess the concentration of the inocula, and the associated load (and proportion) of each serotype in the spiked samples was determined. Samples containing one serotype were inoculated with low numbers of pneumococci (i.e., they had loads similar to those of “minor” serotypes in samples with more than one serotype; see below); however, the serotype contained within them represented 100% of the pneumococcal content. As such, the viable count data from samples with one serotype were included when calculating the mean load of the minor type, but excluded when calculating the mean percent of the minor serotypes.

Spiked samples consisted of medium alone (*n* = 4) or contained one, two, three, or four serotypes (*n* = 7, 38, 26, and 6, respectively). Samples inoculated with pneumococci contained a mean of 2.4 serotypes (95% CI: 2.2, 2.6), with a mean load of 2.11 × 10^5^ colony-forming units (CFU)/ml (95% CI: 1.68 × 10^5^, 2.55 × 10^5^). Samples inoculated with more than one serotype contained a “major type” (the serotype with the highest percent abundance in each sample) and one or more “minor types” (the other serotypes present in each sample). Major types ranged from 67.78% to 98.71% (median 93.69%, interquartile range [IQR]: 88.67, 96.12) of the total pneumococcal content in each sample, with a load ranging from 3.08 × 10^4^ to 5.70 × 10^5^ CFU/ml (median 1.56 × 10^5^, IQR: 5.73 × 10^4^, 3.28 × 10^5^). The minor types ranged from 1.29% to 14.49% (median 4.96%, IQR: 3.05, 6.92) of the total pneumococcal content in each sample, with the load ranging from 6.95 × 10^2^ to 3.89 × 10^4^ CFU/ml (median 8.19 × 10^3^, IQR: 2.95 × 10^3^, 1.85 × 10^4^).

Each set of spiked samples contained randomly selected aliquots shipped frozen on dry ice to the research groups. One set was tested by conventional serotyping (the reference method, see below) in the project laboratory at the Murdoch Childrens Research Institute.

### Field Samples

The 260 nasopharyngeal (“field”) samples were from 260 children aged ≤24 mo in six low- and middle-income countries (Table 2). Samples were collected and stored at ULT using protocols consistent with WHO guidelines [14,17]. The frozen samples were shipped on dry ice to the project laboratory in Melbourne. Samples were dispensed into aliquots and stored as for the spiked samples.

**Table 2:** Field sample information.

Field Site	Number of Swabs	Type of Study, Year of Collection	Age of Children (in Months)	Vaccination Status of Children	Other Information	Type of Swab Tip	Reference, if Applicable
Bangladesh	39	This study, 2011	7–24	Unvaccinated		Cotton	
Fiji	5	Carriage study, 2003–2004	8–19	Unvaccinated		Cotton	
Fiji	48	Vaccine trial, 2006–2007	8–19	Not specified^1^		Cotton	[18]
The Gambia	42	This study, 2010	8–24	Unvaccinated (*n* = 14) or had previously received 1 (*n* = 11), 2 (*n* = 9), or 3 (*n* = 8) doses of PCV7		Alginate/calcium alginate	
Kenya	20	This study, 2008	7–13	Unvaccinated		Rayon	
Kenya	8	Carriage study, 2003–2006	0–24	Unvaccinated		Rayon	[19]
Kenya	5	Carriage study, 2007–2008	12–22	Unvaccinated		Rayon	[20]
Kenya	2	Carriage study, 2000	3–5	Unvaccinated		Rayon	[21]
Papua New Guinea	47	This study, 2010	6–24	Unvaccinated	Children had presented for routine vaccinations or as outpatients at Goroka Hospital; all had cough and most had other respiratory symptoms such as breathing difficulties, runny nose, or eye discharge	Cotton	
South Africa	44	Vaccine trial, 2006	7–17	Vaccinated with three or four doses of PCV7	Fourteen of the children were HIV positive	Dacron polyester	[22]

All swabs were nasopharyngeal, except for those from Papua New Guinea, which were pernasal.

^1^Vaccination status was known to original investigators, but was not provided as part of this study.

### Reference Serotyping Method

Conventional methods were used to identify and serotype the pneumococci carried in the samples. Sample aliquots were thawed and mixed, and 50 μl was used to conduct a viable count by serial dilution and plating on selective medium (HBA plates containing 5 μg/ml gentamicin). After 36–44 h of incubation at 35–37°C in 5% CO_2_, up to 120 well-separated alpha-haemolytic colonies were randomly selected. To do this, the culture plate was evenly divided into eight sections, and a section was chosen from a previously prepared random list. The outermost colony in the designated sector was then picked and designated the “first” colony. The remaining colonies on the whole plate were then picked. If not all of these colonies were required, sections were prioritised from a list of randomly generated numbers. If not all the colonies in a particular section were required, the operator selected colonies from the outer edge of the plate working towards the centre. Throughout the random colony selection process, only well-separated alpha-haemolytic colonies were chosen. An example of each morphological variant was also subcultured if not chosen during random selection. Selected colonies were then subcultured onto HBA plates. Subcultured colonies underwent pneumococcal identification using optochin, and then bile and Phadebact Pneumococcus (MKL Diagnostics) tests, as appropriate. Pneumococcal isolates were serotyped by Quellung reaction and/or latex agglutination (see below). Latex agglutination was conducted using the Denka Seiken kit [23] according to the manufacturer’s instructions [24], except that we used 15 μl of reagent for each test. If all reactions were negative, testing was repeated using 30 μl. For the 72 tests not included in the Denka Seiken kit (seven types [13, 37, 42, 43, 44, 45, and 48] and 65 factors), we used latex reagents prepared in house [25,26] using antisera from Statens Serum Institut (SSI) Diagnostica. A negative control reagent was prepared using normal rabbit serum (Antibodies Australia). Pneumococcal isolates were serotyped by Quellung reaction [27,28] using polyclonal antisera from the SSI.

The primary colony (i.e., the first randomly selected colony for which a serotype was obtained) was serotyped with both the Quellung reaction and latex agglutination. Subsequent colonies were serotyped with latex agglutination, using the minimum number of reactions needed to confirm whether the serotype was the same. Any different serotypes detected were fully serotyped by latex agglutination and Quellung reaction. Laboratory staff were fully blinded during sample processing and participated in an external quality assurance program during the course of the project (RCPA Quality Assurance Program; http://www.rcpaqap.com.au).

When used to examine spiked samples, the reference method had no false positives and four false negatives (serotypes 14 and 23F were not detected once, and 6B not detected twice), all occurring when the target was present as a minor serotype.

### Alternate Serotyping Methods—Overview

To identify serotyping methods for testing, we conducted literature reviews and contacted pneumococcal researchers, gave presentations at international meetings to explain the project, and circulated a project fact sheet. We identified and contacted 29 research groups. Fifteen research groups joined the study. Of the remaining 14 groups, 11 had methods that were no longer in use, had been superseded by other approaches, or were considered by the investigators to be unsuitable for detecting multiple serotype carriage. Three research groups were unable to participate because of funding constraints, existing work commitments, or an inability to reach agreement on the terms of the material transfer agreement (each *n* = 1).

Fifteen research groups participated in this study, testing 20 different methods, which were numbered on enrolment into the study (Table 3). Five groups tested both direct (i.e., testing from sample aliquot) and culture-based (i.e., with an initial broth- or agar-based amplification step) versions of their method. All 20 methods were tested using the spiked sample aliquots. Frozen samples were sent to each research group for testing. Researchers used up to 50 μl and were blinded during sample testing. Following analysis of the spiked sample results (see below), five methods were selected to test the field sample aliquots and were sent aliquots for blinded testing as described above.

**Table 3:** Key characteristics of alternate pneumococcal serotyping methods.

Type of Method	Method Number^1^	Direct Detection or Culture Amplification	Method Technology and Description	Number of Serotypes Detected			Level of Quantification	Other Analyses	Reference
				Total	Individually	As Part of a Group^2^			
**Genotypic**									
	1	Direct detection	mPCR	58	35	23	Semi-quantitative	None	[29]
	11	Culture amplification	mPCR	41	32	9	Qualitative	None	[30]
	16	Culture amplification	mPCR	54	22	32	Qualitative	None	[30–32]
	5	Direct detection	mPCR/reverse line blot hybridisation	68	33	35	Qualitative	None	[33–36]
	10	Culture amplification	Restriction fragment length polymorphism of *ply*NCR (non-coding region), followed by mPCR and Quellung serotyping	32	27	5	Qualitative	Presence of co-colonisation	[30,37]
	19	Direct detection	PCR detected by electrospray ionisation mass spectrometry	59	26	33	Semi-quantitative	MLST results	[38,39]
	22	Culture amplification	As for method 19	59	26	33	Semi-quantitative	MLST results	[38,39]
	6	Culture amplification	mPCR and microarray	42	12	30	Qualitative	None	[40]
	15	Direct detection^3^	Microarray	93	53	40^3^	Quantitative (relative abundance)	Detection of antibiotic resistance genes, determination of genetic relatedness^4^	[13,41]
	4	Culture amplification^3^	As for method 15	93	53	40^3^	Quantitative (relative abundance)	Detection of antibiotic resistance genes, determination of genetic relatedness^4^	[13,41]
	7	Direct detection	Multiplex real-time PCR	32	28	4	Semi-quantitative	None	[42]
	20	Culture amplification	As for method 7	32	28	4	Semi-quantitative	None	[42]
	14	Direct detection	Real-time PCR^5^	47	20	27	Semi-quantitative	None	[43]
	21	Culture amplification	As for method 14^5^	47	20	27	Semi-quantitative	None	[43]
	12	Direct detection	Sequetyping, single PCR, and sequencing	30	26	4	Qualitative	None	[44]
	13	Culture amplification	As for method 12	30	26	4	Qualitative	None	[44]
**Phenotypic**									
	9	Culture amplification	Latex broth, latex agglutination from broth culture	72	8	64	Qualitative	None	[45]
	18	Culture amplification	Latex sweep, latex agglutination from a sweep of colonies	91	89	2	Qualitative	None	[46]
	8	Direct detection	Multiplex immunoassay with heat-kill step	23	23	0	Qualitative	None	[47]
	17	Direct detection	Antigen capture assay	16	12	4	Semi-quantitative	None	[48]

^1^Methods 2 (a variant of method 10) and 3 were assigned a number but did not participate in the study and are not included in the table.

^2^Some methods detect closely related serotypes as part of a group rather than individually (e.g., 18B/C versus 18B or 18C).

^3^Methods 4 and 15 can also be analysed to the level of the individual call for 93 serotypes (see Methods).

^4^Also detects a subset of other bacterial species.

^5^The method was updated prior to testing of field samples to detect 58 total serotypes (18 individually and 40 as part of a group).

mPCR, multiplex PCR.

### Alternate Serotyping Methods—Detailed Methodologies

All incubations were carried out at 35–37°C in ~5% CO_2_ unless otherwise indicated.

#### Method 1 (direct multiplex PCR)

A subset of 22 spiked samples was processed with this method. Initially, 25 μl of sample was spun in a microcentrifuge at 5,220*g* for 2 min. The supernatant was discarded, 200 μl of phosphate-buffered saline (PBS) added, and the pellet resuspended. DNA was extracted using the QuickGene DNA tissue kit S with the QuickGene Mini 80 apparatus (Fuji Film Corporation) and eluted in 200 μl of buffer CDT. Then, 10 μl of extracted DNA was used as a template for multiplex PCR (mPCR) reactions. Each reaction was performed in a total volume of 50 μl using AmpliTaq Gold DNA Polymerase with GeneAmp (Applied Biosystems), and amplified in a Veriti Thermo Cycler (Applied Biosystems) for 40 cycles. PCR products were detected using a Beckman CEQ 8000 genetic analyser system. Primers were labelled for recognition of the product in the CEQ 8000 analyser as green or blue peaks and by size (141 to 515 base pairs). *LytA* PCR was used to detect pneumococcal DNA in the sample as previously described [29], except that primers were labelled and products were detected using the CEQ 8000. Samples were tested in four multiplex assays: (1) five primer pairs to detect five serotypes, (2) 12 primer pairs to detect 13 serotypes, (3) five primer pairs to detect 23 serotypes, and (4) five primer pairs to detect 31 serotypes. When assays performed with primer pairs that detect more than one serotype were positive, additional PCRs using specific primers for the serotypes in the group were conducted.

#### Method 11 (culture mPCR)

In this method, 50 μl of sample was plated onto a selective blood agar plate (containing 5% sheep blood and 5 μg/ml gentamicin) and incubated overnight. DNA was extracted from the entire culture plate using a DNeasy Blood & Tissue Kit (Qiagen). Approximately 500 ng of extracted DNA was then used for each of seven subsequent mPCR reactions [30] using *Taq* polymerase produced in house. If the DNA was positive for *cpsA*, the *cps* primers were omitted and the reaction repeated to avoid competition of these primers with primers for minor serotypes that may be present. A similar approach was taken for any positive serotyping reactions.

#### Method 16 (culture mPCR)

Initially, 50 μl of sample was plated onto Columbia III agar with 5% sheep blood (BD) and incubated overnight. DNA was then extracted from all the plate growth using a DNeasy Blood & Tissue Kit (Qiagen). All seven mPCR reactions were then applied to each sample, using 5 ng of DNA as template. To detect the minor serotypes, the seven mPCR reactions were repeated, using 100 ng of DNA. PCR reactions and primer sequences were as previously described [30–32].

#### Method 5 (direct mPCR/reverse line blot)

DNA was extracted from 50 μl of sample using the NucliSENS easyMAG total nucleic acid extractor kit (bioMérieux) or the GenElute Mammalian Genomic DNA Miniprep Kit (Sigma-Aldrich). DNA was eluted in 110 μl, and 10 μl used for each subsequent test. Initially, *lytA* PCR [49] was performed to screen for the presence of pneumococci. *LytA*-positive samples were then tested by mPCR/reverse line blot (RLB) assay to identify serotypes, as previously described [33,34]. All positive samples were tested with both membranes. If serogroup 6 was detected, serotype-specific PCR [35] was conducted. When isolates were available, the Quellung reaction was performed to distinguish closely related serotypes. Samples that were positive for *lytA*, but non-typeable by mPCR/RLB, were tested by *cpsA-B* PCR and sequencing [36].

#### Method 10 (culture restriction fragment length polymorphism)

In this method, 50 μl of sample was streaked onto a Columbia sheep blood agar plate and incubated overnight. DNA was extracted from a loopful of the bacterial lawn using the QIAamp DNA Mini Kit (Qiagen). The entire lawn was then harvested from the primary culture agar plate and stored at −80°C. PCR amplification of *ply*NCR was performed using 31.4 μl of extracted DNA. To identify co-colonisation, restriction fragment length polymorphism (RFLP) analysis of the *ply*NCR PCR amplicon was performed on the extracted DNA, as described previously [37]. Latex agglutination or mPCR was performed on aliquots that had a single or multiple serotypes detected by RFLP, respectively. mPCR was conducted using 31.4 μl of extracted DNA, similarly to Pai et al. [30] except that PCR products were analysed with the Agilent bioanalyser.

#### Method 19 (direct PCR/electrospray ionisation mass spectrometry)

In this method, 50 μl of sample was diluted 1:4, lysed by bead beating, and treated with proteinase K at 56°C for 15 min, and DNA was extracted using the KingFisher DNA extraction instrument (Thermo Scientific), as previously described [38]. Then, 5 μl of extracted DNA was used for each of eight PCR reactions. The PCR primers targeted pneumococcal serotype and MLST genotyping markers [39]. Following PCR amplification, a fully automated electrospray ionisation mass spectrometry (ESI-MS) analysis was performed using the PLEX-ID PCR/ESI-MS research platform. Base compositions were compared to a database derived from the sequences of known organisms and to signatures from reference standards [39].

#### Method 22 (culture PCR/ESI-MS)

In this method, 1 μl of sample was cultured on a sheep blood agar plate and incubated for 48–72 h. Growth was then harvested using a needle, and boiled for 15 min at 95°C. Twenty-nine of the 81 spiked samples tested did not grow even when 20 μl was cultured, so DNA was directly extracted from ~30 μl of original sample by boiling lysis. Extracted DNA was then subjected to PCR and ESI-MS as outlined for method 19.

#### Method 6 (culture mPCR and microarray)

Initially, 50 μl of sample was cultured on a fresh rabbit blood agar plate for 24 h. Growth was harvested for DNA extraction and analysis, as previously described [40]. In brief, microarrays were created by spotting oligonucleotide probes onto a glass slide using a SpotArray 7.2 (PerkinElmer). A two-step mPCR (to amplify the gene of interest and then label the PCR products) was conducted in two batches each. The resultant products were hybridised to a microarray slide for 16 h before being scanned (GenePix personal 4100A, Axon Instruments), and the signal intensity calculated with GenePix Pro 6.0.

#### Method 4 (culture microarray)

In this method, 45 μl of the neat sample, or of two 10-fold serial dilutions, was spread on selective agar plates (colistin sulphate, oxolinic acid, blood agar; Oxoid) and incubated overnight. For the plate with the highest density of distinct non-confluent colony growth, all colonies were scraped into 1 ml of sterile PBS. DNA extractions were performed using the QIAamp DNA Mini Kit (Qiagen), including lysis buffer (20 mM Tris/HCl, 2 mM EDTA, 1% v/v Triton, 20 mg/ml lysozyme) and RNase treatment as previously described [13]. DNA was eluted in ≤200 μl of H_2_O and quantified using a NanoDrop spectrophotometer. Approximately 300 ng of the DNA was fluorescently labelled with either ULS-Cy3 or ULS-Cy5 using the Agilent Technologies Genomic DNA ULS Labeling Kit. The fluorescently labelled samples were then hybridised overnight to the BμG@S SP-CPS v1.4.0 microarray, according to the Agilent Array CGH protocol. Following hybridisation, the microarrays were washed and scanned, and intensity data acquired using an Agilent microarray scanner and feature extraction software. The statistical calls of which serotypes, or combination of serotypes, were present in the sample, together with the relative abundance of each serotype, were determined using Bayesian-based algorithms [41].

#### Method 15 (direct microarray)

This method was performed as per method 4, except that DNA was extracted from 50 μl of sample using a NucleoSpin Tissue XS DNA isolation kit (Macherey-Nagel) and eluted in 20 μl of H_2_O. Whole genome amplification was performed on 10 μl of extracted DNA using the GenomePlex Whole Genome Amplification Kit (Sigma). The amplified DNA was cleaned up using an Amicon Ultra 0.5 ml (Millipore) centrifugal filter and quantified using a NanoDrop spectrophotometer. Note that a subset of 16 spiked samples was processed with this method.

#### Method 7 (direct multiplex real-time PCR)

DNA was extracted from 50 μl of sample using the easyMAG automatic extraction system (bioMérieux) and eluted in 100 μl. The multiplex real-time PCR [42] was performed in eight tubes (each containing four fluorophores) targeting 29 pneumococcal *cps* regions and the *lytA* gene. Then, 5 μl of extracted DNA was added to each tube and reactions were performed using the Bio-Rad CFX96 real-time PCR system.

#### Method 20 (culture multiplex real-time PCR)

This method was performed as per method 7, except that the 50 μl of sample was inoculated into 8 ml of Todd Hewitt broth (Sigma-Aldrich) (with 0.5% yeast extract) and incubated overnight. Then, 0.5 ml of the overnight culture was used for DNA extractions as in method 7.

#### Method 14 (direct real-time PCR)

DNA was extracted from 20 μl of sample using the QIAmp DNeasy Blood & Tissue Kit (Qiagen). Only samples positive for *lytA* by quantitative real-time PCR were included in serotyping analysis. Real-time PCR was performed as previously described using 6 μl of extracted DNA for each reaction using 33 sets of primers/probes [43]. If no increase in fluorescent signal was observed after 40 cycles, the sample was deemed negative for that serotype. Note that after testing of the PneuCarriage samples was complete, new primers and a probe were designed for serotype 35F/34 to improve the specificity of this assay (forward: 5′-CGAATTCGGAAARCAATGTGTTT-3′, reverse: 5′-TATGCAATTTAGCTGCAAAAAATCC-3′, probe: 5′-FAM-TTGACATTTTTCCTCTAGATGGTTAT-TAMRA-3′). The new assay no longer detects serotype 47.

#### Method 21 (culture real-time PCR)

In this method, 10 μl of sample was cultured in 3 ml of Todd Hewitt broth (Biolife Italiana) overnight, and DNA from 200 μl of broth culture was extracted using the QIAmp DNeasy Blood & Tissue Kit (Qiagen). Testing was then conducted as in method 14.

#### Method 12 (direct sequetyping)

DNA from 50 μl of sample was extracted by heat lysis [44], the debris pelleted, and the supernatant diluted 1:10. Sequetyping was performed as previously described [50]. In brief, a region spanning the *cpsB* gene was amplified by PCR (using 2 μl of extracted DNA per reaction), the amplicon purified, and the nucleotide sequence determined. The nucleotide sequences were used to interrogate a publically available gene database (GenBank), and the match with the greatest nucleotide homology indicated the serotype. If co-colonisation was detected, the amplicons were subcloned into a plasmid vector, cloned, and re-sequenced. If a sample was *cpsB* negative, the PCR was repeated using 10 μl of template. A subset of 29 spiked samples was tested with this method.

#### Method 13 (culture sequetyping)

This method was conducted as per method 12, except that the 50 μl of sample was inoculated into 10 ml of Brain Heart Infusion (Oxoid) broth. After 18–24 h of incubation, cells from 8 ml of broth were pelleted by centrifugation, resuspended in 50 μl of H_2_O, heat lysed, and processed as above.

#### Method 9 (culture latex broth)

In this method, 25 μl of sample was added to 3 ml of serum broth (SSI Diagnostica) and incubated overnight. The culture was then serogrouped and/or typed using the commercially available Pneumotest-Latex kit as recommended by the manufacturer (SSI Diagnostica) [45]. Each culture was tested against all the A-I and P-T pools. Further differentiation required the use of group and/or factor serum (SSI Diagnostica) [45].

#### Method 18 (culture latex sweep)

Initially, 10 μl of sample was cultured on a selective agar plate (Columbia CNA agar with 5% sheep blood) overnight. A suspension of all the colonies was made and tested as described previously [46]. In brief, 10 μl of suspension and 10 μl of latex reagent were mixed on a glass slide, rocked for up to 2 min, and observed for agglutination and clearing of the background. Testing was done following the SSI antisera typing scheme, testing each sample against all the pools and then following up with appropriate group, type, and factor reactions. Pneumococci were provisionally identified as non-typeable if there was weak agglutination with pool B and serogroup 19 latex antisera but no agglutination with group 19 factor antisera (19b, 19c, 19f, 7h). During spiked sample testing, method 18 had five false-positive results for serotype 13 and reported a non-typeable pneumococcus that was not present in 27/81 (33%) spiked samples. For testing field samples, the method was updated to improve specificity of serotype 13 (confirmation with Quellung reaction), and non-typeables were not reported. Note that if scanty growth was present on the overnight plate, the method was repeated using up to 40 μl of sample.

#### Method 8 (direct multiplex immunoassay)

Samples were heat-killed by incubation at 60°C for 45 min, and assayed as described previously [47]. In brief, samples and a positive control were diluted 1:5 in an absorbent buffer (containing CPS and 10A) and added to a filter-bottomed microtitre plate. Carboxylated Luminex microspheres (previously conjugated with pneumococcal polysaccharides) were added to the plate, followed by diluted 89-SF (US Food and Drug Administration) as the antibody source. After a 20min incubation, the plate was washed twice using PBS with 0.05% Tween 20, and an anti-human IgG-*R*-phycoerythrin conjugate was added to the plate. After another 20 min, the plate was washed as before and read on a Bio-Plex reader (Bio-Rad) to generate a mean fluorescent intensity (MFI) reading for each antigen. Positive samples were indicated by a 30% or greater reduction in sample MFI compared to the positive control MFI.

#### Method 17 (direct antigen capture assay)

In this method, 50 μl of sample was processed by centrifugation at 16,000*g* for 2 min and the supernatant diluted 1:2 in PBS. Following this, 25 μl was applied in duplicate to the Luminex immunoassay, performed as previously described [48]. In brief, the diluted sample was mixed with xMAP beads (covalently conjugated to specific monoclonal antibodies for the serotypes and C-polysaccharide) and assayed on the Luminex 100/200 platform. Positive reactions were detected using a mixture of polyclonal serogroup-specific antibodies (SSI) and an anti-rabbit *R*-phycoerythrin fluorescent conjugate.

### Identification of the Best Serotyping Method

For both the spiked and field sample testing, our primary analyses were the percent sensitivity and the PPV of each method. Sensitivity was defined as the percentage of serotypes present in samples that were correctly identified by each method. For spiked samples, sensitivity was calculated for the major serotype, minor serotype(s), and overall. For field samples, sensitivity was calculated for samples containing a single serotype, for samples with multiple serotypes (as a proxy for major and minor serotypes), and overall. The PPV was defined as the percentage of serotypes identified that were actually present in the samples, i.e., the proportion of identified positives that were true positives.

Sensitivity and PPV were calculated according to the level of discrimination provided by the method. For example, if a method claimed to detect 6A/B, and the sample contained 6B, results reported as 6A/B would be deemed correct. However, if a method claimed to detect 6A and 6B as individual serotypes, and the sample contained 6B, then results reported as 6A would be incorrect. Note that for microarray (methods 4 and 15), some closely related serotypes were reported as a group, with the individual serotype call in brackets (e.g., 6A/B [6B]). In this case, results were analysed both to the level of the group and to the level of the individual serotype call. For simplicity of analysis, if a method did not claim to detect a serotype (e.g., 23F) but the sample contained that serotype, this result was deemed incorrect. Pneumococci identified as non-typeable were excluded from all analyses. Serotypes 15B and 15C were analysed as 15B/C, as these serotypes can interconvert [51].

For the spiked samples, results were compared with the inocula. The result 19A or 19F was deemed correct for strain PMP843, which types as 19F by phenotypic methods but has genetic characteristics of 19A (similar to Pimenta et al. [52]). We also evaluated the accuracy of the microarray data (from method 4) in determining the percent relative abundance of each serotype present in a sample, the ability of method 10 (culture RFLP) to detect co-colonisation, and the ability of methods 14 and 21 (direct and culture real-time PCR) to determine pneumococcal load and semi-quantitative serotype-specific load.

Selection of methods for field sample testing was primarily based on scientific performance in the testing of the spiked samples, using a cutoff of ≥70% sensitivity to detect minor serotypes and ≥90% PPV. Methods that met these criteria were also assessed on other aspects (see below), specifically the number of serotypes that they were currently capable of detecting (individually or as part of a group) and the perceived ease of incorporating additional serotypes. This approach was used to ensure that methods were able to serotype carriage isolates (which may be more diverse than invasive isolates) and were also capable of detecting the emergence of non-vaccine serotypes in the post-PCV era. Methods were also assessed for their potential for quantification. Using these criteria, five methods progressed to testing of field samples.

For field samples it was more challenging to differentiate false from true positives, because the exact sample contents were unknown, and a highly sensitive method would likely detect serotypes that other methods did not. Results from the reference serotyping method, research group methods, and additional testing of discrepant results were used to create a “study gold standard”, against which the performance of the alternate serotyping methods was assessed.

To determine the study gold standard, we examined results for each sample, and considered a serotype to be a true positive if it was found by (1) the reference method and one or more alternate methods or (2) three or more alternate methods or (3) two methods that covered both phenotypic and genotypic approaches (e.g., microarray and latex sweep). Note that methods 14 and 21 (direct and culture real-time PCR assays, respectively) were considered to be one method for these analyses. Where a serotype was found by only one method or by two methods of similar approach (i.e., two genotypic or two phenotypic assays), this was deemed a discrepant result. Field samples with discrepant results were further investigated by testing of sample aliquots by single-plex quantitative real-time PCR [53] and/or serotyping of the stored isolate (if obtained during the reference method testing) by Quellung reaction (Microbiological Diagnostic Unit, University of Melbourne).

### Statistical Analysis

Statistical tests were performed using GraphPad Prism version 5.04 for Windows, (GraphPad Software). Quantitative data were analysed with the D’Agostino and Pearson omnibus normality test, and reported as mean (95% CI) or median (IQR) accordingly. Spearman’s correlation was used to examine the relationship between variables.

## Results

### Spiked Samples

The reference serotyping method (conventional serotyping) had 100%, 96%, and 98% sensitivity to detect the major, minor, and overall serotype populations, respectively. No false positives were identified (PPV 100%).

**Fig 1:**
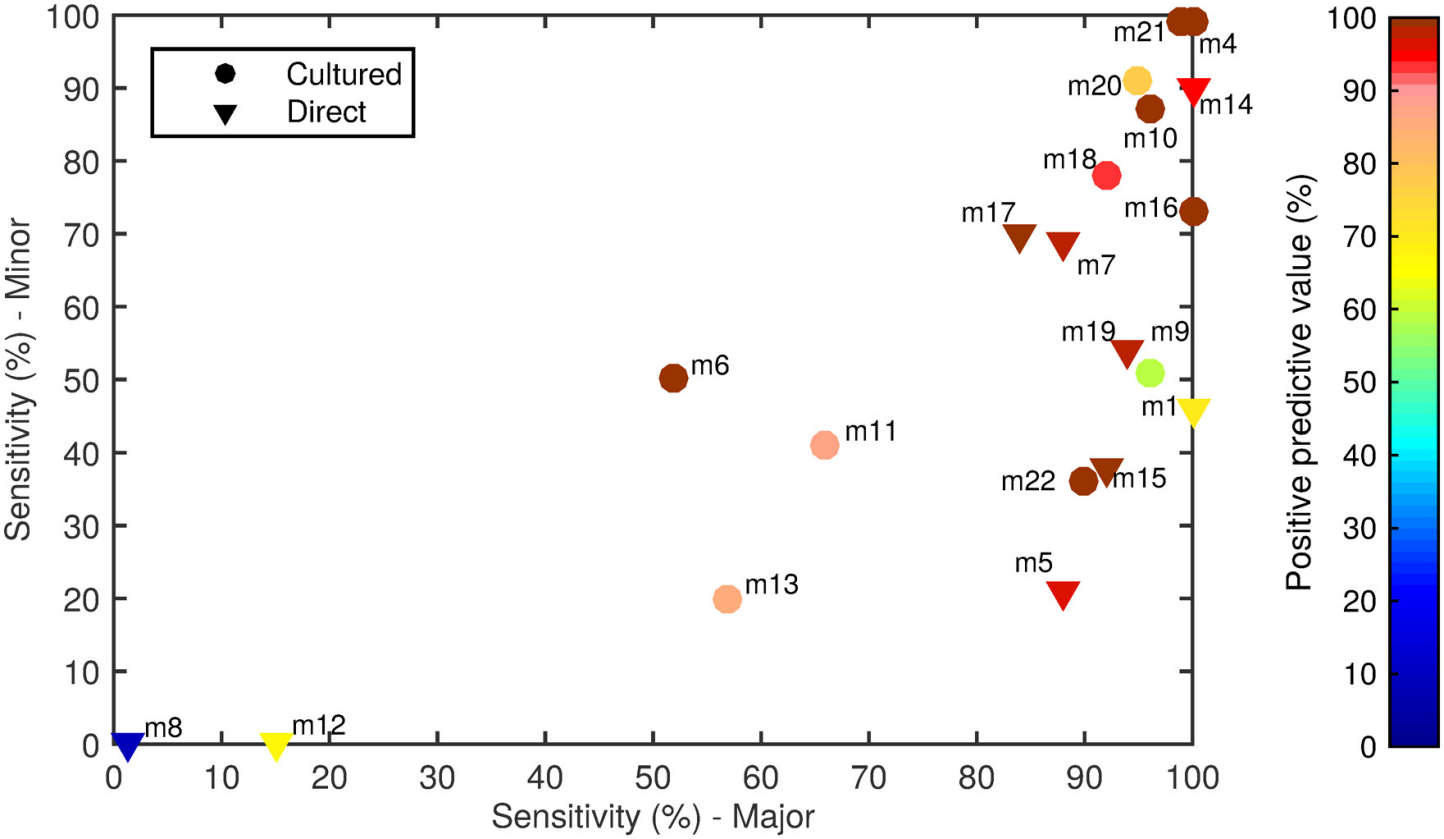
Spiked sample testing results. For each method (labelled m1–m22), the sensitivity of detection of the major serotypes (*x-*axis) and minor serotypes (*y-*axis) is plotted on the graph, with the PPV shown in colour according to the colour bar on the right. Methods that directly tested the sample or included a culture amplification step are represented by triangles and circles, respectively.

The 20 alternate serotyping methods had overall sensitivities ranging from 1% to 99%, with similar ranges for the major and minor serotype populations. The PPV ranged from 8% to 100% (Fig 1; Table 4; S1 Data). Some methods performed well overall, but encountered problems with particular serotypes. For example, method 7 (direct multiplex real-time PCR) did not detect serotype 4, method 11 (culture mPCR) did not detect serotype 9V and had poor specificity for 23F, and method 9 (culture latex broth) had several false-positive reactions with two tests (one test detecting serotypes 13 and 28, and the other detecting serogroup 16 and serotypes 36 and 37).

**Table 4:** Performance of alternate serotyping methods when testing spiked samples.

Type of Method	Method Number	Direct Detection or Culture Amplification	Method Technology and Description	Sensitivity (95% CI)^1^			PPV (95% CI)^1^	Key Performance against Initial Screen of ≥70% Sensitivity for Minor Serotypes and ≥90% PPV
				Major Serotype	Minor Serotype	Overall		
**Genotypic**								
	1	Direct detection	mPCR	100 (81, 100)^2^	46 (28, 66)	67 (52, 80)	69 (53, 82)	Low sensitivity and PPV
	11	Culture amplification	mPCR	66 (55, 77)	41 (31, 51)	51 (44, 59)	87 (79, 93)	Low sensitivity and PPV
	16	Culture amplification	mPCR	100 (95, 100)^2^	73 (64, 81)	84 (78, 89)	100 (98, 100)^2^	
	5	Direct detection	mPCR/RLB hybridisation	88 (79, 95)	21 (14, 30)	49 (42, 57)	96 (90, 99)	Low sensitivity
	10^3^	Culture amplification	RFLP of *ply*NCR region, followed by mPCR and Quellung serotyping	96 (89, 99)	87 (79, 93)	91 (86, 95)	99 (97, 100)	
	19	Direct detection	PCR/ESI-MS	94 (85, 98)	54 (44, 63)	70 (63, 77)	98 (94, 100)	Low sensitivity
	22	Culture amplification	PCR/ESI-MS	90 (81, 95)	36 (27, 46)	58 (51, 66)	100 (97, 100)^2^	Low sensitivity
	6	Culture amplification	mPCR and microarray	52 (40, 63)	50 (40, 60)	51 (43, 58)	99 (94, 100)	Low sensitivity
	15^4^	Direct detection^5^	Microarray	92 (64, 100)	38 (15, 65)	62 (42, 79)	100 (78, 100)^2^	Low sensitivity
	4^3^ ^,^ ^6^	Culture amplification	Microarray	100 (95, 100)^2^	99 (95, 100)	99 (97, 100)	100 (98, 100)^2^	
	7	Direct detection	Multiplex real-time PCR	88 (79, 95)	69 (59, 77)	77 (70, 83)	97 (93, 99)	Low sensitivity
	20	Culture amplification	Multiplex real-time PCR	95 (87, 99)	91 (84, 95)	92 (88, 96)	78 (72, 84)	Low PPV
	14^3^	Direct detection	Real-time PCR	100 (95, 100)^2^	90 (83, 95)	94 (90, 97)	95 (91, 98)	
	21^3^	Culture amplification	Real-time PCR	99 (93, 100)	99 (95, 100)	99 (96, 100)	99 (96, 100)	
	12	Direct detection	Sequetyping, single PCR, and sequencing	15 (4, 34)	0 (0, 9)^2^	6 (2, 15)	67 (22, 96)	Low sensitivity and PPV
	13	Culture amplification	Sequetyping, single PCR, and sequencing	57 (45, 68)	20 (13, 29)	36 (29, 43)	85 (75, 92)	Low sensitivity and PPV
**Phenotyptic**								
	9	Culture amplification	Latex broth, latex agglutination from broth culture	96 (89, 99)	51 (41, 61)	70 (63, 76)	58 (52, 65)	Low PPV
	18^3^	Culture amplification	Latex sweep, latex agglutination from a sweep of colonies	92 (84, 97)	78 (69, 85)	84 (78, 89)	93 (88, 96)	
	8	Direct detection	Multiplex immunoassay with heat-kill step	1 (0, 7)	0 (0, 3)^2^	1 (0, 3)	8 (0, 36)	Low sensitivity and PPV
	17	Direct detection	Antigen capture assay	84 (74, 92)	70 (61, 79)	76 (69, 82)	100 (97, 100)^2^	

^1^Calculated from results of testing 81 spiked samples, except for methods 1 (direct mPCR), 12 (direct sequetyping), and 15 (direct microarray), which tested 22, 29, and 16 spiked samples, respectively.

^2^These are one-sided 97.5% confidence intervals, as they have been clipped at one tail.

^3^Selected to test the field samples.

^4^When method 15 was analysed to the level of the individual serotype call, it had 92% sensitivity for the major serotype, 19% sensitivity for the minor serotypes, 52% overall sensitivity, and 100% PPV.

^5^Following whole genome amplification.

^6^When method 4 was analysed to the level of the individual serotype call, it had 100% sensitivity for the major serotype, 95% sensitivity for the minor serotypes, 97% overall sensitivity, and 100% PPV.

When spiked samples were tested with method 4 (culture microarray), only one serogroup 6 serotype was reported (e.g., 6A/B [6B]) for three samples containing both serotypes 6A and 6B, so the sensitivity and PPV were slightly lower when results were analysed to the level of the individual serotype call (Table 4). The same was true for method 15 (direct microarray).

Seven methods met our initial criteria of ≥70% sensitivity to detect minor serotypes and ≥90% PPV when testing the spiked samples (Table 4). Method 16 (culture mPCR) had similar performance to the real-time PCR assays. However, the real-time PCR assays have the potential to provide quantitative data and so were selected over method 16, which is a qualitative assay (PCR products visualised on a gel). Method 17 (direct antigen capture assay) performed well but was capable of detecting only 16 serotypes, so was not selected. The remaining five alternate serotyping methods were tested using the 260 field samples (Table 5).

**Table 5:** Performance of alternate serotyping methods when testing field samples.

Type of Method	Method Number	Direct Detection or Culture Amplification	Method Technology and Description	Sensitivity (95% CI)^1^			PPV (95% CI)^1^
				Samples with 1 Serotype	Samples with ≥2 Serotypes	Overall	
**Genotypic**							
	10	Culture amplification	RFLP of *ply*NCR region, followed by mPCR and Quellung serotyping	87.8 (81.8, 92.4)	65.7 (57.3, 73.5)	77.5 (72.4, 82.1)	96.4 (93.2, 98.3)
	4^2^ ^,^ ^3^	Culture amplification	Microarray	97.5 (93.8, 99.3)	93.7 (88.4, 97.1)	95.8 (92.8, 97.7)	93.9 (90.7, 96.3)
	14^2^	Direct detection	Real-time PCR	75.5 (68.1, 81.9)	72.7 (64.7, 79.8)	74.2 (68.9, 79.0)	89.4 (84.9, 92.9)
	21^2^	Culture amplification	Real-time PCR	79.1 (72.1, 85.1)	81.1 (73.7, 87.2)	80.1 (75.1, 84.4)	82.2 (77.4, 86.4)
**Phenotypic**							
	18	Culture amplification	Latex sweep, latex agglutination from a sweep of colonies	81.7 (74.9, 87.3)	77.6 (69.9, 84.2)	79.8 (74.9, 84.2)	91.4 (87.4, 94.5)

^1^Results of testing 260 field samples calculated against the study gold standard (see main text for definition).

^2^Only 259 samples tested (one sample tube empty upon arrival).

^3^Method 4 was occasionally incorrect for serogroup 11. When analysed to the level of the individual serotype call, it had 95.8% sensitivity for samples with one serotype, 90.9% sensitivity for samples with ≥2 serotypes, 93.5% overall sensitivity, and 91.7% PPV.

### Field Samples

**Fig 2:**
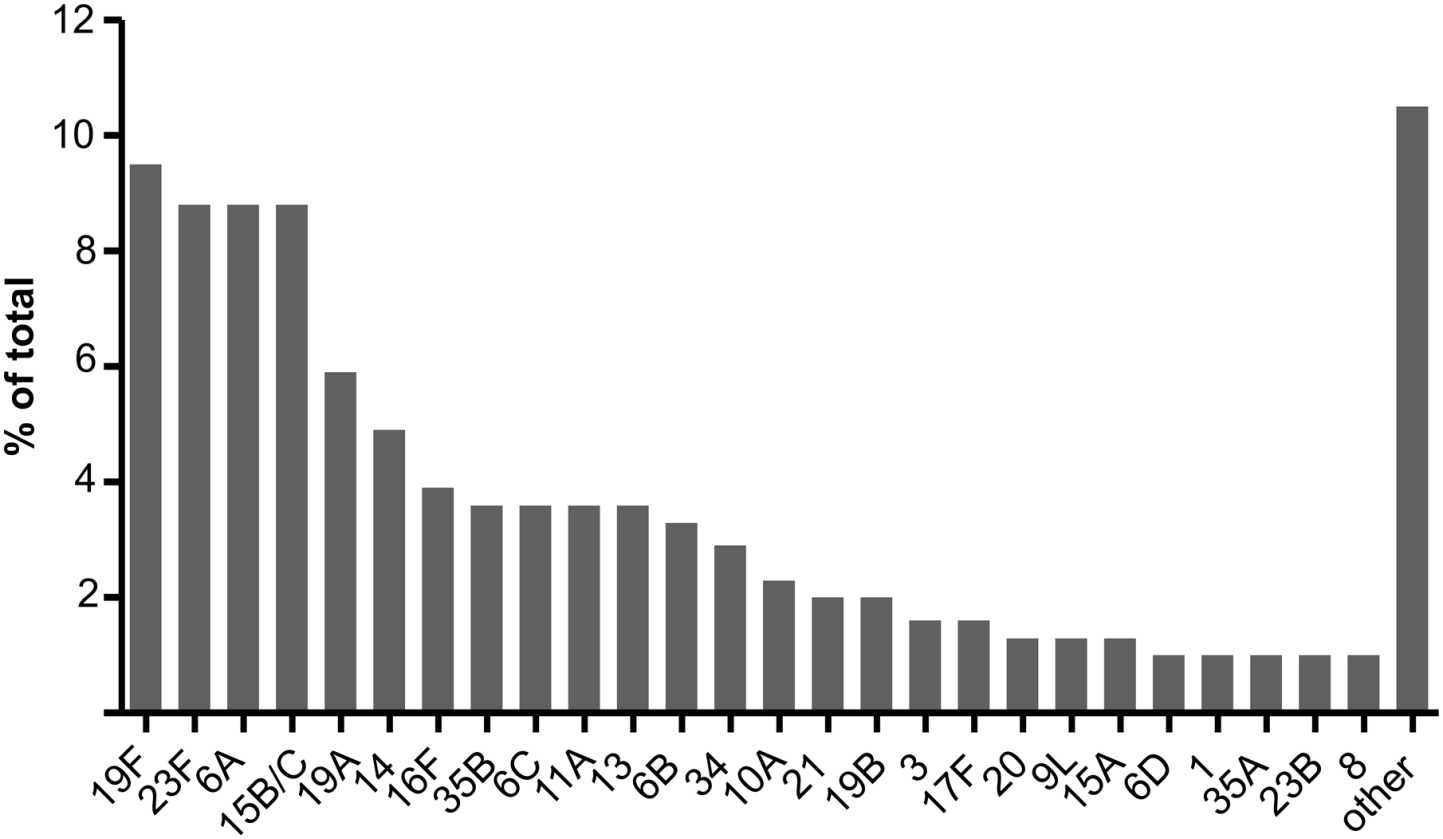
Serotype distribution in field samples. A total of 307 serotypeable pneumococci (representing 49 serotypes) were identified in 260 nasopharyngeal swab samples collected from children in six countries. The 26 most common serotypes are shown here, with the remaining 23 serotypes identified combined as “other”.

The 260 field samples contained 307 serotypeable pneumococci. Forty-nine of the known 97 serotypes, including twelve of the PCV13 types, were identified at least once. PCV7, PCV10, and PCV13 types represented 27.1%, 29.0%, and 45.6% of the 307 serotypeable pneumococci, respectively. Serotypes 19F, 23F, 6A, 15B/C, 19A, 14, 16F, 6C, 11A, 13, and 6B were the most common (Fig 2; S3 Data) and together accounted for 68% of the total. There were 35, 164, 44, 14, two, and one samples that contained zero, one, two, three, four, and five serotypes, respectively. Note that samples containing “zero” serotypes included those from which no pneumococci or only non-typeable pneumococci were isolated. Samples that contained pneumococci had a median of 1.0 (IQR: 1.0, 2.0) serotypes and contained multiple serotypes in 27.1% of cases. Using the reference method, 234 samples resulted in growth of alpha-haemolytic colonies (consistent with pneumococci), with a mean load of 3.75 × 10^5^ CFU/ml (95% CI: 2.37 × 10^5^, 5.14 × 10^5^). The mean relative abundance of minor serotypes (calculated from a subset of 39 field samples containing multiple serotypes and no non-typeables) was 18.7% (95% CI: 14.5%, 22.9%). When testing the 260 field samples, the reference serotyping method had 96.3% sensitivity for samples containing one serotype, 90.9% sensitivity for samples containing multiple serotypes, and 93.8% sensitivity overall. The PPV was 99.6% (one false positive).

**Fig 3:**
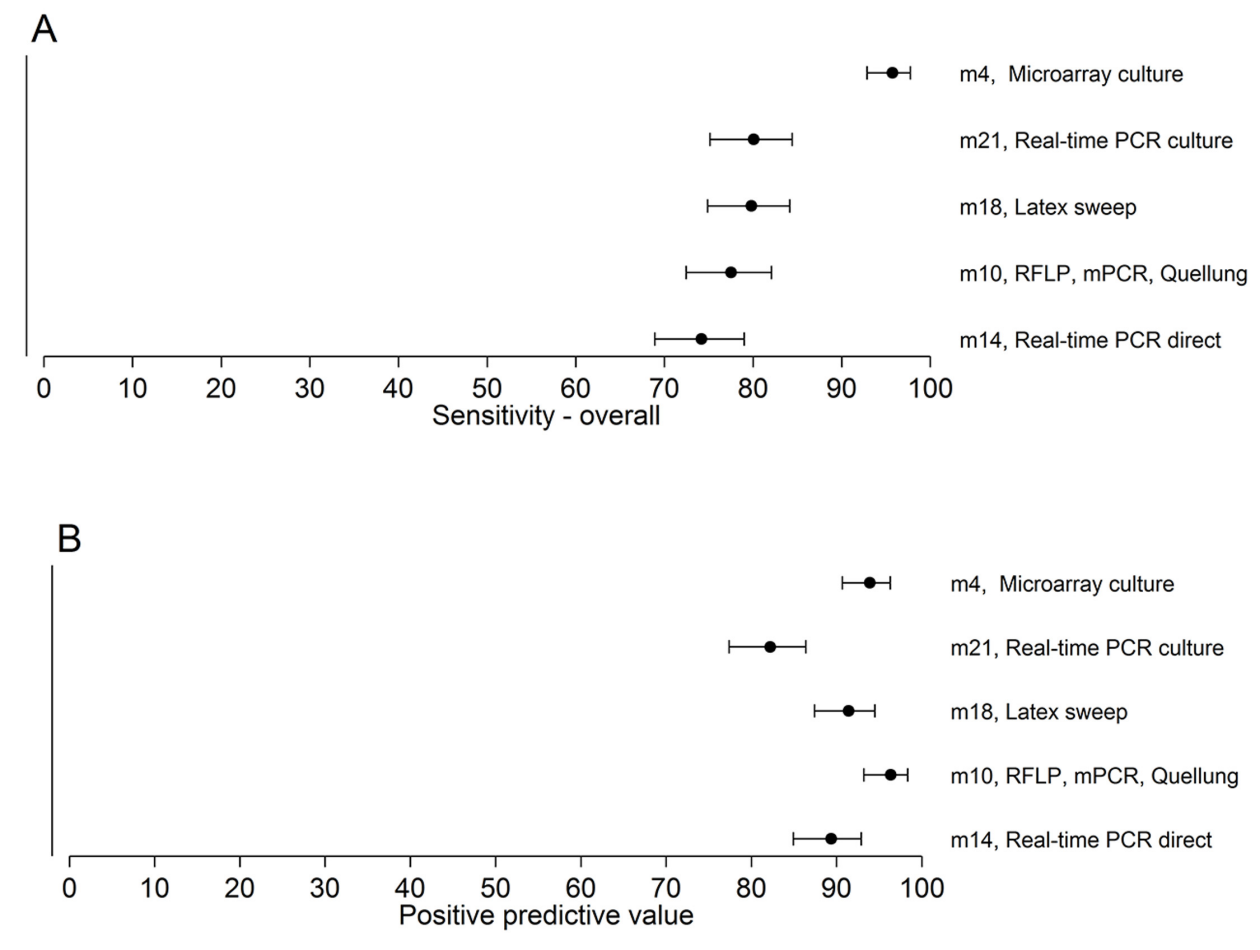
Sensitivity and PPV of the five methods testing the 260 field samples. The point estimates and 95% CIs for sensitivity (A) and PPV (B) are depicted. The sensitivity of method 4 is higher than those of the other methods.

For the five methods used to test field samples, the resultant sensitivities for samples containing one serotype, samples containing multiple serotypes, and overall ranged from 75.5% to 97.5%, 65.7% to 93.7%, and 74.2% to 95.8%, respectively. The PPVs ranged from 82.2% to 96.4% (Table 5; Fig 3; S2 Data).

### Secondary Analyses

**Fig 4:**
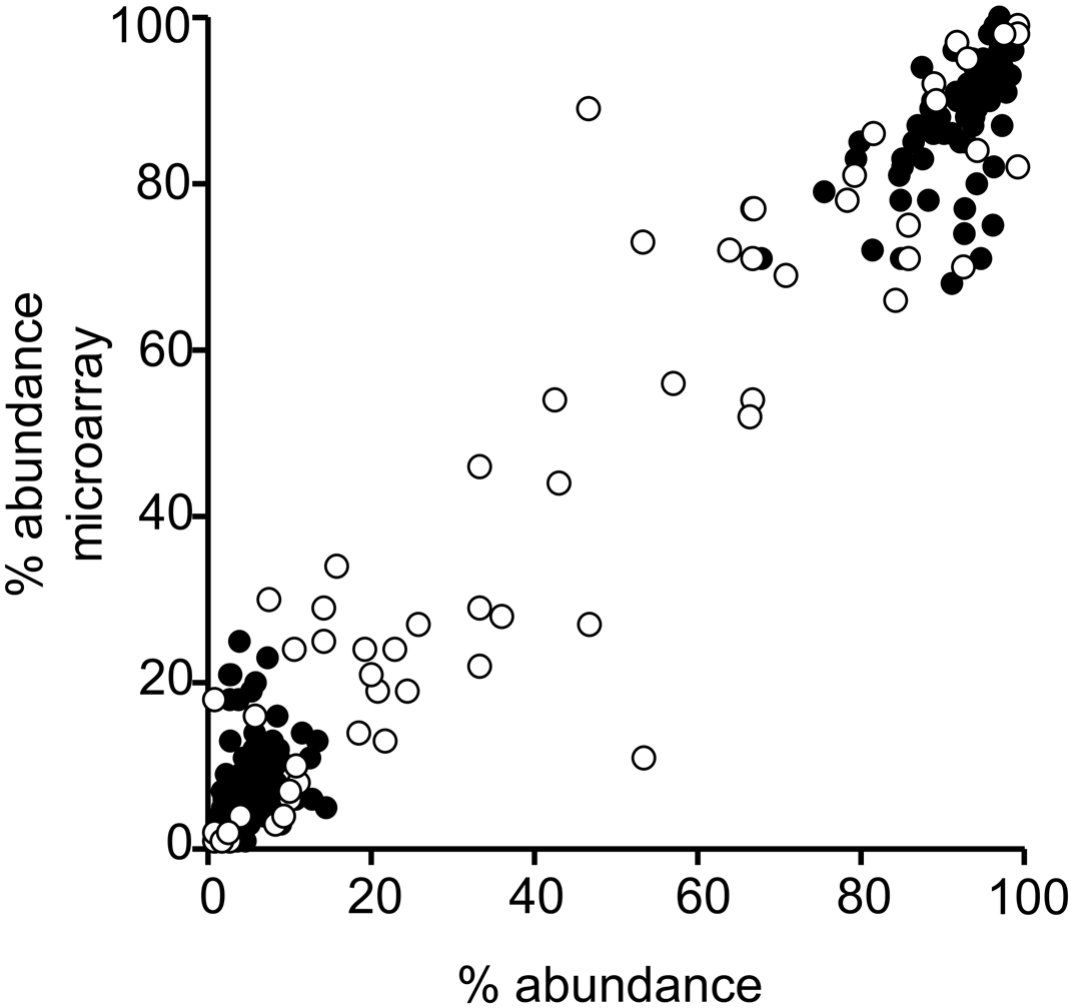
Performance of microarray in determining percent abundance of serotypes in spiked and field samples. The percent relative abundance reported by method 4 (culture microarray) compared with the inocula for 174 serotypeable pneumococci within 70 spiked samples with multiple serotypes (filled circles) and compared with results obtained by conventional serotyping according to the reference method for 61 serotypeable pneumococci within 27 field samples with multiple serotypes (open circles). For the spiked samples, the correlation of relative abundance results between the inocula and microarray was significant (*p <* 0.001): Spearman’s *r* = 0.863 (95% CI: 0.818, 0.897). Similarly, the correlation between actual relative abundance and microarray results was significant for the field samples (*p <* 0.001): Spearman’s *r* = 0.907 (95% CI: 0.847, 0.944).

For methods 4 (culture microarray), 10 (culture RFLP), 14 (direct real-time PCR), and 21 (culture real-time PCR), we conducted secondary analyses. We evaluated the ability of method 4 (culture microarray) to provide accurate serotype-specific relative abundance for each serotype present within a sample (Fig 4; S4 Data). For the spiked samples, the relative abundance results from method 4 were compared to the inocula (Fig 4; S4 Data) using the 70 spiked samples that contained multiple serotypes (70 samples containing 174 serotypeable pneumococci). The median difference in relative abundance between the inocula and microarray results was 3.0% (IQR: 1.4%, 5.3%). For the field samples, microarray results for relative abundance were compared to the reference method for a subset of samples (*n* = 27) that contained multiple serotypes, had consistent serotyping results for both methods, and did not contain any non-typeables (Fig 4; S4 Data). The 27 samples contained 61 serotypeable pneumococci. The median difference in relative abundance between the reference method and microarray results was 5.3% (IQR: 1.2%, 12.7%). Results were closely correlated for spiked samples (*p <* 0.001; Spearman’s *r* = 0.863 [95% CI: 0.818, 0.897]) and field samples (*p <* 0.001; Spearman’s *r* = 0.907 [95% CI: 0.847, 0.944]).

Method 10 (culture RFLP) contains a screen for co-colonisation that indicates whether multiple isolates of pneumococci are present, which might be useful as a screening test for multiple serotype carriage. For spiked samples, the ability of this method to detect co-colonisation was assessed against the inocula and had 90% sensitivity and 100% specificity. For field samples, the co-colonisation screen was assessed against the study gold standard and had 44.3% sensitivity and 65.8% specificity. Specificity results should be interpreted with caution, as samples that contain a single serotype may contain multiple strains of that serotype with different RFLP profiles. The ability of the quantitative real-time PCR method 14 to quantify pneumococcal loads was determined using the spiked samples, with the finding that the estimated loads were higher than, but significantly correlated with, the inocula (S1 Fig; S4 Data). We also determined the impact of bacterial load and sample complexity on method performance and the ability of methods 14 and 21 to quantitate pneumococcal loads and provide semi-quantitative data on serotype loads (S1 Text).

## Discussion

The PneuCarriage project was a multi-centre comparative study designed to identify the best pneumococcal serotyping methods in order to support future carriage studies and to facilitate monitoring of pneumococcal vaccine impact in resource-poor settings. Five methods were selected for testing nasopharyngeal samples based upon their performance serotyping laboratory-prepared samples. Method 4 (culture microarray) had the best performance overall.

The performance of individual pneumococcal serotyping methods was highly variable. When 20 serotyping methods were evaluated using 81 laboratory-prepared (“spiked”) samples, 13 failed to meet our performance criteria of ≥70% sensitivity to detect minor serotypes and ≥90% PPV. Although this raises concerns about the performance of these methods and the validity of some previous studies, it is important to note that many of these methods performed well when testing pure cultures and/or identifying the major serotype, often reflecting the original purpose of the assays. Although some of the methods we investigated may be appropriate for diagnostic use, we did not test their suitability for such a purpose.

Some methods (e.g., method 17) performed well but were not selected as they were capable of detecting only a smaller subset of the 97 known serotypes. This is of concern for monitoring serotype replacement and PCV impact as rare serotypes may emerge and become more common, particularly with the introduction of higher valency vaccines. Some methods had particular technical issues. For example, it is likely that the heat-kill step in method 8 (direct multiplex immunoassay) greatly diminished its sensitivity. Although we did not fully unblind the research groups (to enable future use of the reference samples), details on “problem” serotypes were provided to facilitate optimisation of the methods.

Given that performance in spiked sample testing was a critical component in assessing methods in this study, spiked samples were constructed to reflect nasopharyngeal samples in terms of their overall pneumococcal load, as well as in having a range of serotypes, including representatives of serotypes that are common or rare in carriage. Conventional serotyping was used to underpin development of the “study gold standard”, as the Quellung reaction and latex agglutination methods employed are recommended by the World Health Organization [14]. To thoroughly characterise the samples, we randomly selected up to 120 colonies from each sample, giving >99% power to detect a minor serotype of 5% abundance.

Based on scientific performance and technical aspects of the methods, we selected five methods to test the 260 nasopharyngeal (“field”) samples. Although this study was not designed to survey the serotypes carried in children, the serotype diversity and distribution results from the field samples (Fig 2) are generally consistent with carriage studies performed in paediatric populations [54]. Samples were derived from both vaccinated and unvaccinated individuals, and included a substantial proportion of non-vaccine types relevant in testing the applicability of these methods in PCV-vaccinated settings. All five methods had a PPV and overall sensitivity of >82% and >76%, respectively, in the field sample testing. In contrast to its performance in the spiked sample testing, method 10 (culture RFLP) had poor sensitivity to detect co-colonisation in the field samples, effectively ruling out its use as a screening test. Methods 14 and 21 (direct and culture real-time PCR) had a large number of false-positive results (27 and 53 false positives, respectively) when testing the field samples, including 19 false positives for the assay detecting serotypes 35F, 34, and 47F. The primer and probe sequences for this assay have subsequently been updated with the aim of improving specificity (see Methods).

The reasons for the differences between the spiked and field sample results were not explored, but may include the increased biological complexity of the field samples, such as the presence of cells and nucleic acids from other microorganisms and the host. This finding is important as it indicates that spiked samples alone are insufficient to properly assess method performance. Previous studies have found that using a culture amplification step increases sensitivity of detection [55,56], and our findings were consistent with this. However, culture amplification increased the number of false positives for multiplex real-time PCR. Caution should be applied when using non-selective culture amplification steps in combination with sensitive molecular methods, in line with recent findings that other streptococci can possess capsular gene sequences and thereby confound some pneumococcal serotyping assays [57]; this phenomenon may have contributed to the higher number of false positives detected by method 21 when testing the field samples. Direct molecular methods that do not require a culture step may be particularly useful in settings with suboptimal sample storage conditions, high antibiotic use, or other factors that may affect pneumococcal viability, as remaining pneumococcal DNA could provide important epidemiological information. Such methods would require thorough evaluation to ensure that they can discriminate molecular signatures of pneumococci from those present in closely related species.

The performance of individual pneumococcal serotyping methods was highly variable, and there was also considerable variation within a particular type of technology (e.g., mPCR). As such, it is important that establishment of any pneumococcal serotyping method is supported by appropriate training and a rigorous quality system framework. As exemplified by the “19A-like” 19F isolate used in the spiked samples, serotyping results may occasionally differ depending on whether genotypic or phenotypic methods are employed [52,58]. This highlights the importance of continued method validation and quality control, particularly when genotypic methods based on a single gene target are employed. As it is anti-capsular antibodies that confer vaccine-induced immunity against pneumococci, phenotypic methods are ultimately more relevant in cases where genotypic results do not correlate with capsule structure.

The microarray (method 4) is able to detect all known serotypes and accurately measure their percent relative abundance and is suitable for high-throughput testing. Furthermore, the method can determine the genetic relatedness of isolates, and these results could be deposited in a global database [59]. However, it is unlikely that microarray will be practical for in-country testing in resource-poor countries. Method 10 requires establishment of three separate techniques and may therefore be challenging to implement in low-income settings, whereas method 14 or 21 would be a practical approach given that real-time PCR assays are widely used. Method 18 (culture latex sweep), which also performed well, has been successfully applied in low-income settings [46,60]. The ability of latex sweep and microarray to detect multiple serotypes was in line with a previous comparison study [13]. None of the methods tested were truly quantitative. However, microarray (method 4) accurately measured proportions of serotypes in both spiked and field samples, and could potentially be combined with a quantitative measure of total pneumococcal carriage (e.g., *lytA* quantitative real-time PCR [61]) to determine serotype-specific densities.

Although this study is, to our knowledge, the most comprehensive of its kind to date, not all published pneumococcal serotyping assays were tested [62,63]. Some published serotyping methods that were not tested in our study include the MassTag method [64], the combined multiplex immunoassay and PCR assay [63], and the US Centers for Disease Control and Prevention (CDC) mPCR and real-time PCR methods [30,62]. The CDC methods were similar to methods 11 and 16 (mPCR) and methods 7, 20, 14, and 21 (real-time PCR) in our study, and the majority of primers used in the combined multiplex immunoassay and PCR assay were from method 5 or the CDC. Although the MassTag method and the combined multiplex immunoassay and PCR assay have been applied to a small number of complex samples (nasopharyngeal samples and lung aspirates [64] and pleural fluid samples [65]), neither has undergone comprehensive validation for this purpose. Of note, Carvalho et al. found that serotype-specific PCR and other sequencing-based assays may be confounded by the presence of non-pneumococcal species, particularly in some sample types [57]. Given that we found that the performance of similar methods varied markedly, and that methods optimised for one application (such as testing pure isolates or invasive samples) did not necessarily perform well when testing more complex samples, the performance of currently available or future serotyping methods not included in this study should be examined using blinded testing of the PneuCarriage samples or a similar well-characterised collection. Overall, our findings underscore the importance of conducting rigorous comparative testing of pneumococcal serotyping methods before their application in carriage studies.

Another limitation of our study is that we did not include detection of non-typeable pneumococci, which are commonly carried in some populations [66,67], in our analysis. Additionally, we did not separately assess the various DNA extraction and culture methods used to prepare samples for serotyping. We also assessed the performance of the methods in only one laboratory each, and so cannot make conclusions about other variables such as reproducibility, inter-operator variability, and the ability of these methods to be transferred into less-experienced laboratories or resource-poor settings.

The aim of this study was to identify the best pneumococcal serotyping methods for carriage studies. Since the commencement of this study, there has been a growing realisation of the importance of pneumococcal carriage studies as a means of monitoring the impact of existing pneumococcal vaccines, evaluating community (herd) immunity to pneumococci, and evaluating new pneumococcal vaccines. Studies are now in place to use pneumococcal carriage as a proxy for herd immunity, providing a means by which the impact of different PCV schedules can be evaluated. Developers of new pneumococcal vaccines are relying on demonstrated impact on carriage as an important step in vaccine development. Our findings can now be applied to many of the open questions in the field—including whether carriage data (including detection of minor serotype populations) are useful in predicting serotype replacement—or to understanding the emergence of epidemic serotypes. Recent studies have explored whether nasopharyngeal carriage samples can be used to diagnose pneumococcal pneumonia (which is particularly difficult in children) or examine the severity of pneumonia [68–71]. Employing optimal methods for serotyping nasopharyngeal samples from pneumonia patients may further expand these areas of research.

The effect of PCVs on multiple serotype carriage is unclear [72,73], and the utility of assessing the effect of vaccination on carriage for newly emerging vaccines as a “go/no go” point in vaccine development and/or licensure holds promise but remains to be established. We envisage that these methods will now be applied to vaccine impact studies in low-income settings, measuring changes in carriage before and after vaccine introduction in community carriage surveys and/or in children with pneumonia, and monitoring community carriage as a sensitive indicator of herd immunity under various vaccine schedules and levels of coverage. The resultant data, together with data and models from countries with invasive disease surveillance programs, can be used to evaluate vaccine impact and modify vaccination schedules in resource-poor settings.

## Supporting Information

S1 Data Spiked sample data used to calculate sensitivity and PPV. (XLSX)

S2 Data Field sample data used to calculate sensitivity and PPV. (XLSX)

S3 Data Data used to calculate the number of serotypeable pneumococci and the percent represented by each serotype. (XLSX)

S4 Data Data used to examine the ability of method 4 to quantify relative abundance (compared to inocula for spiked samples and compared to the reference method for field samples) and the ability of method 14 to quantify pneumococcal loads. (XLSX)

S1 Fig Pneumococcal loads as determined by *lytA* quantitative real-time PCR compared to inocula for spiked samples. The pneumococcal loads of 77 spiked samples determined by *lytA* quantitative real-time PCR (method 14, genome equivalents/ml) were compared with the loads of the inoculum (CFU/ml). The correlation between the inocula and pneumococcal loads determined by real-time PCR was significant (*p <* 0.001): Spearman’s *r* = 0.800 (95% CI: 0.698, 0.870). Four spiked samples that did not contain pneumococci were excluded from this analysis.
(TIF)

S1 Text Additional results from secondary analyses. (DOCX)

## Abbreviations

CDC: US Centers for Disease Control and Prevention
CFU: colony-forming units
ESI-MS: electrospray ionisation mass spectrometry
HBA: horse blood agar
IQR: interquartile range
MFI: mean fluorescence intensity
MLST: multilocus sequence typing
mPCR: multiplex PCR
PBS: phosphate-buffered saline
PCV: pneumococcal conjugate vaccine
PPV: positive predictive value
RFLP: restriction fragment length polymorphism
RLB: reverse line blot
SSI: Statens Serum Institut
STGG: skim milk-tryptone-glucose-glycerol
ULT: ultra-low temperature
