# Supplementary figures and images for: The PneuCarriage Project: A Multi-Centre Comparative Study to Identify the Best Serotyping Methods for Examining Pneumococcal Carriage in Vaccine Evaluation Studies
Source: PLoS Med. 2015 Nov 17;12(11):e1001903. doi: 10.1371/journal.pmed.1001903 (PMC4648509; doi:10.1371/journal.pmed.1001903)

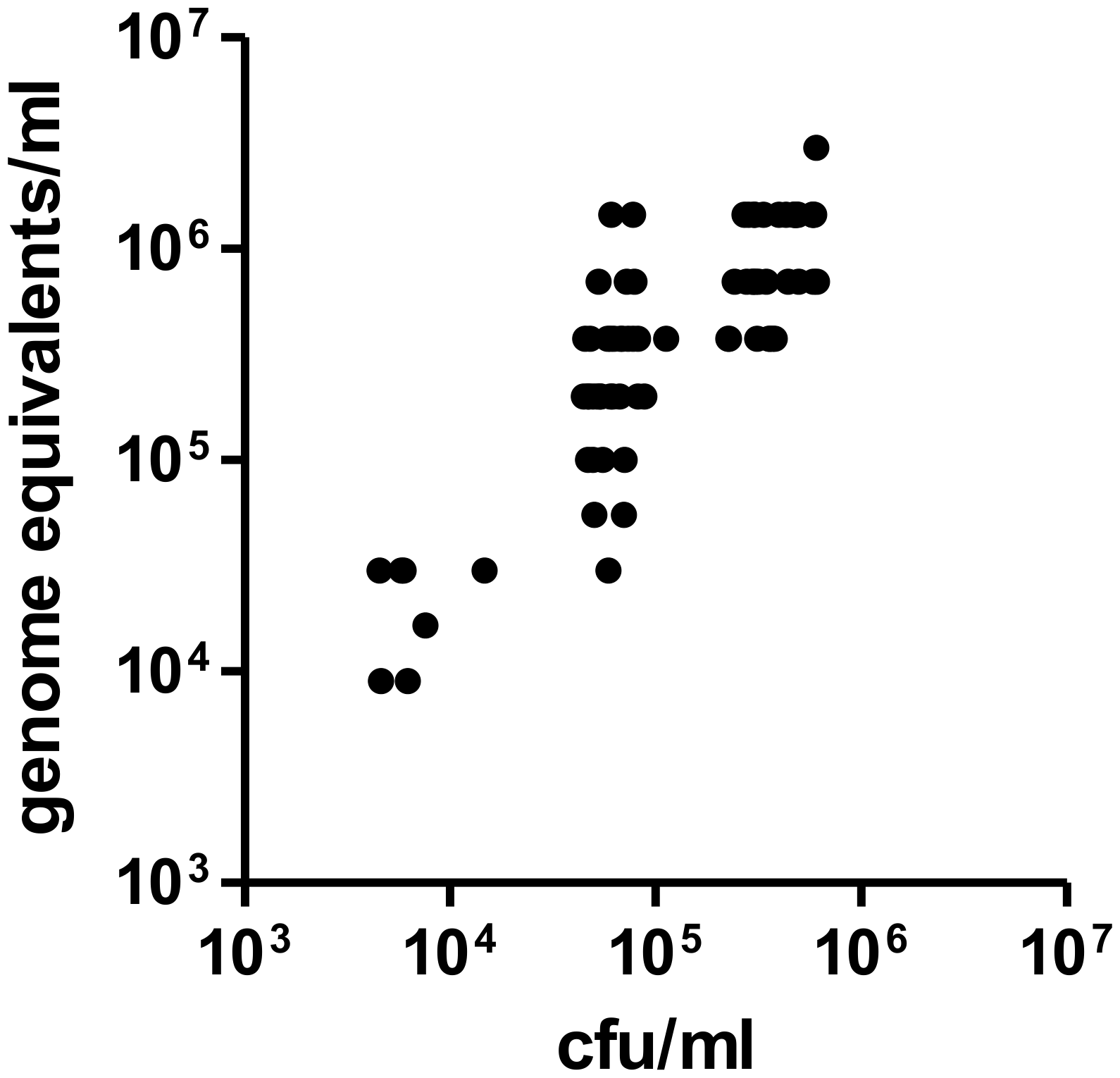

Supplement: S1 Fig — The pneumococcal loads of 77 spiked samples determined by lytA quantitative real-time PCR (method 14, genome equivalents/ml) were compared with the loads of the inoculum (CFU/ml). The correlation between the inocula and pneumococcal loads determined by real-time PCR was significant (p < 0.001): Spearman’s r = 0.800 (95% CI: 0.698, 0.870). Four spiked samples that did not contain pneumococci were excluded from this analysis. (TIF) [file pmed.1001903.s005.tif]
