# Supplementary material for: The PneuCarriage Project: A Multi-Centre Comparative Study to Identify the Best Serotyping Methods for Examining Pneumococcal Carriage in Vaccine Evaluation Studies
Source: PLoS Med. 2015 Nov 17;12(11):e1001903. doi: 10.1371/journal.pmed.1001903 (PMC4648509; doi:10.1371/journal.pmed.1001903)
Supplement: S1 Text — (DOCX) [file pmed.1001903.s006.docx]

## Additional results from secondary analyses

Using the spiked samples, we conducted a preliminary examination of the impact of bacterial load and sample complexity on method performance. Fifteen methods had high sensitivity (defined as ≥70%, mean 91% (95% CI: 84, 99)) for the seven samples with one serotype present. However, the mean sensitivity of these methods was reduced to 65% (95% CI: 50, 80) when detecting minor serotypes. Of note, the sensitivities of method 5 (Direct mPCR/RLB), method 9 (Culture latex broth), method 11 (Culture mPCR), method 13 (Culture sequetyping) and 22 (Culture PCR/ESI-MS) fell below the ≥70% cutoff; most of the sensitivities reduced by more than half. The counts for the seven samples with one serotype present, and for the minor serotypes within the 70 samples with more than one serotype present, were very similar, suggesting that for some methods the sample complexity (i.e. number of serotypes present) was an important factor in reducing sensitivity, rather than just bacterial loads.

In addition, we assessed the ability of methods 14 and 21 (Direct and culture real-time PCR) to quantitate pneumococcal loads and to provide semi-quantitative data on serotype loads. For spiked samples, pneumococcal load data from method 14 (Direct real-time PCR) were compared to the inocula (S1 Figure). The correlation between the inocula and pneumococcal loads determined by quantitative real-time PCR was significant (P < 0.0001): Pearson’s r = 0.727 (95% CI: 0.601, 0.818) and the R^2^ value = 0.529. Pneumococcal loads determined by quantitative real-time PCR were within a log but consistently higher than the inocula: 3.8 x 10^5^ genome equivalents/ ml (IQR: 2.0 x 10^5^,­ 1.1 x 10^6^) compared to 8.2 x 10^4^ CFU/ ml (IQR: 5.4 x 10^4^, 3.6 x 10^5^), P < 0.0001. The median threshold cycle (Ct) value for major serotypes (29.5, IQR: 27.0, 31.0) was lower than those obtained for minor serotypes (33.0, IQR: 32.0 , 35.0), P < 0.0001 (Mann-Whitney test). The median Ct value for false positives was 35.5 (IQR: 29.5, 36.8).

For method 21 (Culture real-time PCR), pneumococcal load data were outside the range of the assay (>10^6^ genome equivalents/ml) and therefore not analysed. The median Ct value for major serotypes (16.0, IQR: 15.0, 18·0) was lower than those obtained for minor serotypes (21·0, IQR: 18.0, ­23.0), P < 0.0001 (Mann-Whitney test). The median Ct value for false positives was 35.5 (IQR: 34.0, ­ 37.0). For field samples, pneumococcal load data were not analysed as they were reported semi-quantitatively for the direct qPCR method and not provided for the culture qPCR method.
